# Influences of Temperature and Time on Habitat Use Patterns of a Semi‐Aquatic Turtle

**DOI:** 10.1002/ece3.72301

**Published:** 2025-11-09

**Authors:** Jena M. Staggs, Donald J. Brown, Madaline M. Cochrane, Andrew F. Badje, Ron A. Moen

**Affiliations:** ^1^ School of Natural Resources and the Environment West Virginia University Morgantown West Virginia USA; ^2^ Pacific Northwest Research Station U.S. Forest Service Amboy Washington USA; ^3^ Department of Ecology Montana State University Bozeman Montana USA; ^4^ Wisconsin Department of Natural Resources Bureau of Natural Heritage Conservation La Crosse Wisconsin USA; ^5^ Natural Resources Research Institute, Department of Biology University of Minnesota‐Duluth Duluth Minnesota USA

**Keywords:** global positioning system (GPS), *Glyptemys insculpta*, habitat selection, movement, telemetry

## Abstract

Many ectothermic vertebrates have predictable seasonal activity and habitat use patterns, but variable patterns at fine temporal scales (e.g., minutes to days) that are likely influenced by thermoregulatory demands on behavior. Wood turtles ( *Glyptemys insculpta* ) are freshwater turtles known to use terrestrial environments during their active period. However, little research has been conducted to quantify the influence of environmental factors on diel habitat use patterns. We used fine‐resolution global positioning system (GPS) tracking and temperature data to quantify wood turtle aquatic‐terrestrial habitat‐use patterns from May through August 2015 and 2016, focusing on the influence of temporal variables and environmental temperature. We found that temporal variables and open canopy air temperature had strong explanatory power for wood turtle aquatic‐terrestrial habitat use patterns, but temperature was a stronger predictor. Terrestrial activity was positively associated with air temperature, resulting in a consistent pattern of daytime terrestrial and nighttime aquatic activity during the pre‐nesting period. Male and female activity patterns diverged during the post‐nesting activity period, with most males returning to the river at night and most females remaining terrestrial, influenced by females moving farther from the river. The results of our study provide valuable information to inform population survey design, habitat management planning, and potential responses to climate change for this unique species of conservation concern.

## Graphical abstract

Reptiles rely on external heat to support metabolic processes, and this reliance could result in predictable habitat use patterns. We compared temperature and time as predictors of aquatic vs. terrestrial habitat use for wood turtles ( *Glyptemys insculpta* ) across hourly to seasonal time scales and found that temperature had stronger explanatory power. Our findings help explain the variability in the timing of activity observed for this species across its geographic distribution and provide useful predictions to support conservation efforts.

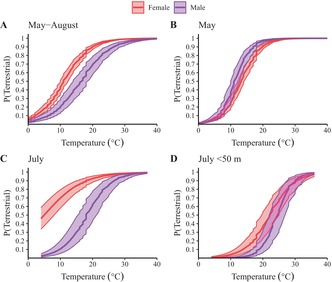

## 1 Introduction

Semi‐aquatic wildlife are uniquely adapted to survive in contrasting environments that provide different resources and environmental conditions (Seymour 1982). For instance, in terrestrial environments, air temperatures can change rapidly depending on vegetation, sun exposure, wind, and humidity, resulting in a wide range of temperatures that vary spatially and temporally across the landscape (Magnuson et al. 1979; Tracy and Christian 1986). In contrast, water buffers temperature changes due to its high specific heat capacity, resulting in lower temperature variances in aquatic environments relative to surrounding terrestrial environments (Boyer 1965; Angilletta 2006). Environmental temperature is an important resource for ectotherms because it directly influences metabolic activity and, consequently, development, growth, survival, and reproduction (Huey 1982; Stevenson 1985; Zuo et al. 2011). Ectotherms often exploit high air temperatures through basking behavior to increase body temperatures, improving the efficiency of physiological processes (Cowles and Bogert 1944; Boyer 1965). Aquatic environments may provide valuable food resources, movement pathways, and refugia from predators or extreme temperatures (Seymour 1982). Thus, environmental temperatures can influence spatiotemporal habitat selection patterns of ectotherms through thermoregulatory behavior (Tracy and Christian 1986; Row and Blouin‐Demers 2006; Fitzgerald and Nelson 2011). Understanding these patterns can inform management actions to improve habitat quality for semi‐aquatic species in thermally limiting environments.

Wood turtles ( *Glyptemys insculpta* ) are semi‐aquatic freshwater turtles endemic to northeastern North America and the Great Lakes Region (Amato et al. 2008). Approximately 82% of their contemporary geographic distribution falls within regions that were glaciated during the Wisconsinan phase of the Pleistocene (Jones et al. 2021), making them one of the most northerly distributed turtles in North America (Ernst and Lovich 2009). Wood turtles require streams for overwintering and use streams throughout their active period (typically April to October) but are also highly terrestrial during the summer months (Kaufmann 1992; Ernst 2001; Brown et al. 2016).

**FIGURE 1:**
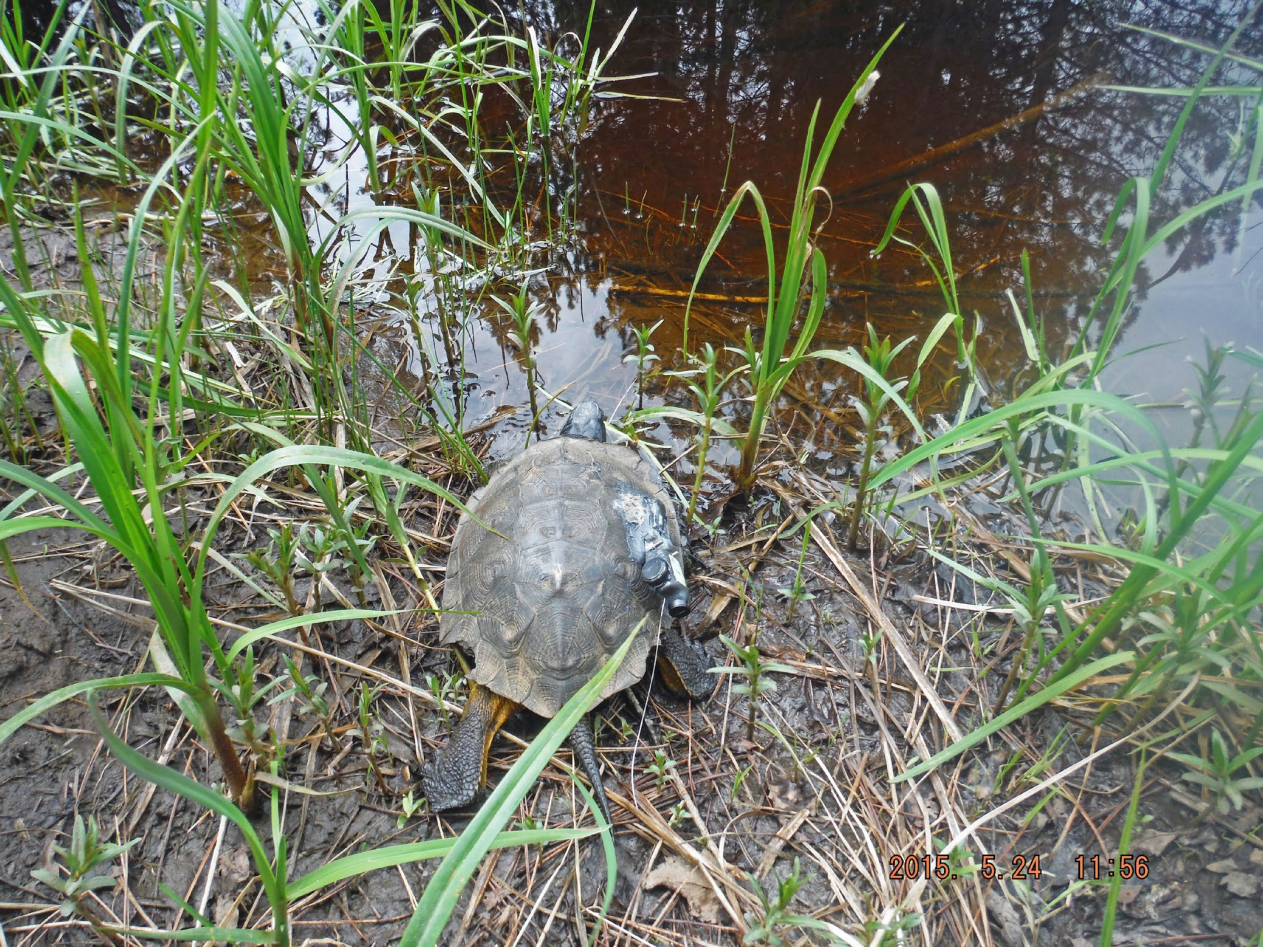
An adult wood turtle ( *Glyptemys insculpta* ) documented moving from land to water during this study of aquatic‐terrestrial habitat use patterns conducted in northeastern Minnesota from 2015 to 2016. Attached to the turtle's carapace are a global positioning system (GPS) tracker (Advanced Telemetry Systems G10 UltraLITE), a very high frequency transmitter (Advanced Telemetry Systems R1680), and an external temperature sensor (Thermochron iButton DS1922L).

It is well documented that wood turtles stay near the river in the spring and fall, regularly moving between riverine and terrestrial environments (Figure 1; Harding and Bloomer 1979; Kaufmann 1992). This behavior is likely motivated by thermoregulatory needs driven by fluctuating temperatures, allowing individuals to exploit terrestrial basking habitats when the air temperature is warm and retreat to the thermally stable aquatic environment during cooler periods (Arvisais et al. 2002; Dubois et al. 2009). However, the pattern is less clear during the summer when temperatures are warmer and less variable. Characterizing habitat use patterns and quantifying the influence of environmental conditions on movement can improve our understanding of habitat requirements and quality for this wildlife species.

Many studies assert increased terrestrial activity and limited use of riverine habitat by wood turtles during the summer (Ernst 1986; Kaufmann 1992; Wallace et al. 2020). Still, there is growing evidence of continued use of riverine habitat, especially by males and at higher latitudes (Dubois et al. 2009; Brown et al. 2016; Waltner 2025). However, our knowledge of wood turtle movement patterns is derived mainly from very high frequency (VHF) radio telemetry studies, which typically are biased towards daytime sampling and usually have temporal resolutions on the order of one location or fewer per day (e.g., Compton et al. 2002; Dubois et al. 2009; McCoard et al. 2016). Recent advances in global positioning system (GPS) tracking technology enable collection of high‐resolution space‐use data for small‐bodied and semi‐aquatic animals such as freshwater turtles (Recio et al. 2011; Christensen and Chow‐Fraser 2014; Seidel et al. 2018). Researchers are beginning to employ this technology to improve our understanding of wood turtle habitat use and selection (Thompson et al. 2018; Cochrane et al. 2019; Latham et al. 2023).

The objective of our study was to estimate and compare the influence of temporal variables and environmental temperature on aquatic‐terrestrial habitat use for adult wood turtles. We used GPS loggers (10‐min fix interval) coupled with temperature loggers placed on each turtle and in multiple environments to link turtle locations to the focal habitat classes and modeled relationships using linear mixed‐effects models. We hypothesized that temperature would have higher explanatory power due to the reliance of ectotherms on external heat for thermoregulation, but that temporal predictors would also perform well due to relationships between time and temperature within days and across seasons.

## 2 Materials and Methods

### 2.1 Study Area

**FIGURE 2:**
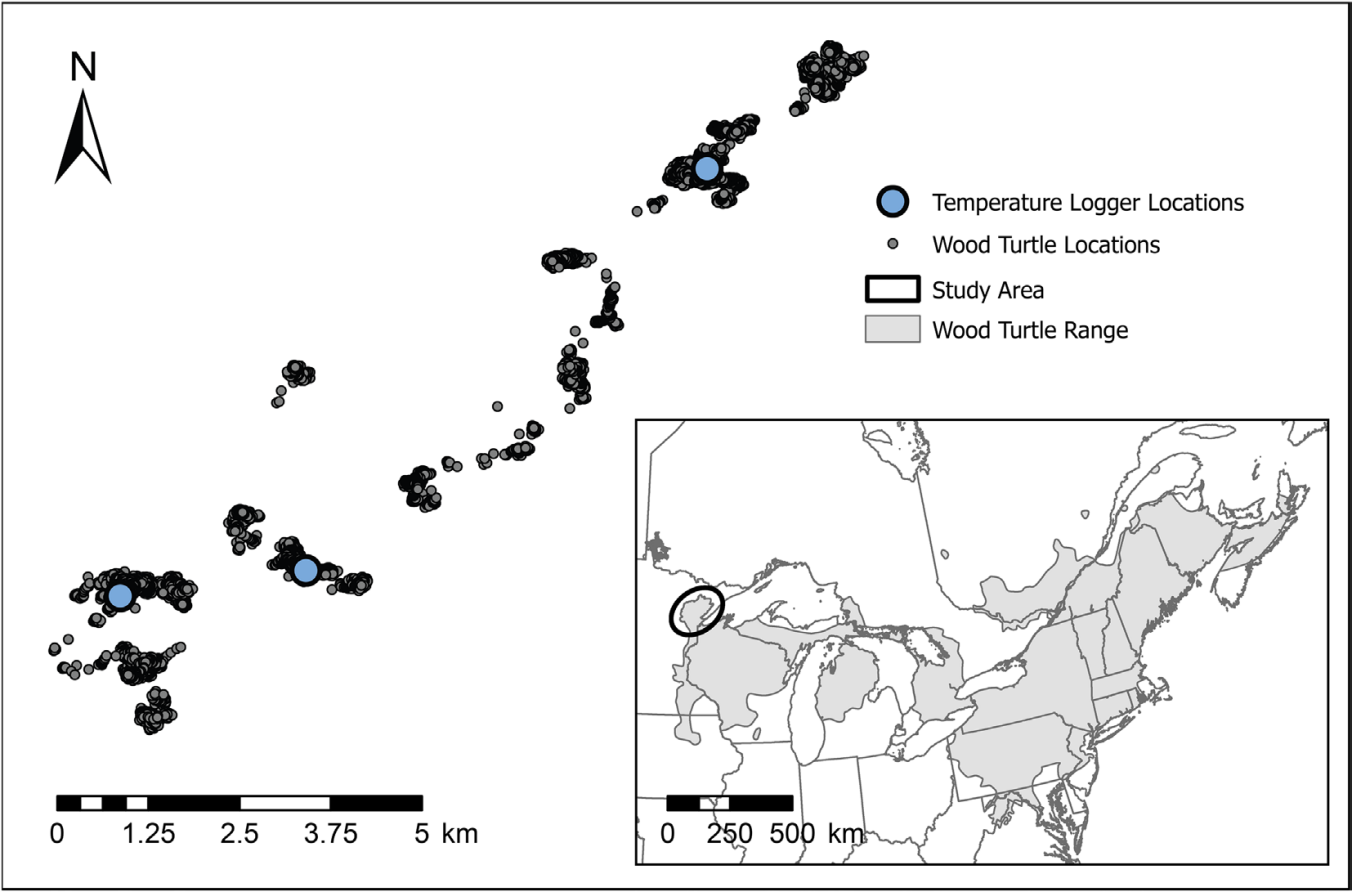
Study area and observation data locations for this study assessing aquatic‐terrestrial habitat use patterns of adult wood turtles ( *Glyptemys insculpta* ) in northeastern Minnesota. Between 2015 and 2016, we used global positioning system (GPS) trackers to record locations of 23 turtles and monitored temperature at each turtle's location and three environmental locations within the study area at 10‐min intervals. At each environmental location, we deployed temperature sensors in the stream and terrestrially in open and closed canopy. The observation points shown here are locations where the GPS units fixed a location.

We tracked individual wood turtles along a 40‐km stretch of river in northeastern Minnesota (Figure 2; specific locations withheld in compliance with the state of Minnesota data practices law). This area is rural and heavily forested, mainly consisting of lowland alder (*Alnus* spp.), aspen (*Populus* spp.), paper birch ( *Betula papyrifera* ), balsam fir ( *Abies balsamea* ), black spruce ( *Picea mariana* ), northern white cedar ( *Thuja occidentalis* ), and pine (*Pinus* spp.; Brown et al. 2016; Cochrane et al. 2019). Approximately 75% of the surrounding watershed is public land (Cochrane et al. 2019).

### 2.1 Data Collection

From May to September 2015 and 2016, we tracked 29 adult wood turtles (carapace length > 17 cm; Harding and Bloomer 1979; Walde et al. 2003) using G10 UltraLITE GPS units (Advanced Telemetry Systems, Isanti, MN, USA) set to collect locations every 10 min. We attached a Thermochron iButton (DS1922L; Maxim Integrated, Dallas, TX, USA) to the carapace of each tracked turtle. We placed environmental temperature loggers at three general locations in the study area (Figure 2). At each location, we recorded open and closed canopy air temperature using Onset HOBO Pendant G receivers (model UA‐004‐64; Bourne, MA, USA) and stream temperature using Thermochron iButtons. All iButton loggers were coated with Plasti Dip (Plasti Dip International, Blaine, MN, USA) to waterproof them (Roznik and Alford 2012), and all temperature loggers were set to collect temperatures every 10 min. For this study, we excluded tracked turtles that lacked temperature data because their iButtons failed, resulting in the inclusion of 23 turtles, including five turtles tracked in 2015 (three females and two males), six turtles tracked in 2016 (four females and two males), and 12 turtles tracked in both 2015 and 2016 (nine females and three males). Female and male midline carapace length ranged from 190 to 213 cm and 207 to 239 cm, respectively. Capture, handling, and study methods were approved by the University of Minnesota Institutional Animal Care and Use Committee (protocol no. 1504‐32514A) and permitted by the Minnesota Department of Natural Resources.

We relocated turtles using VHF transmitters (R1680; Advanced Telemetry Systems) and downloaded GPS data approximately once per month. We specified time information for each observation (i.e., hour, day, month, year). We assigned the observation as night or day based on sunrise and sunset times using the R package suncalc (Thieurmel and Elmarhraoui 2022). In a previous study, Cochrane et al. (2019) improved the location accuracy of the GPS data sets using a moving average approach, and we retained the corrected location data for this study. Cochrane et al. (2019) also developed a data classifier using the temperature and location data and classified each 10‐min observation as aquatic or terrestrial, with 97% of observations classified, which we used for this study.

We calculated the distance to the stream for all terrestrial observations with an associated location. We first created a stream polygon layer in ArcGIS Pro 3.0.1 (ESRI, Redlands, CA) from the National Wetland Inventory using all polygons with a wetland type of “Riverine” (Wilen and Bates 1995), then used the geoprocessing tool “Near” to calculate the distance of each location to the nearest river polygon. For each observation time, we averaged the environmental temperatures across iButton loggers within each habitat class (i.e., stream, open‐canopy terrestrial, closed‐canopy terrestrial). We then screened the air temperature data sets for outliers using bagplots (i.e., bivariate boxplots) and hourly temperature data from a nearby weather station (Rousseeuw et al. 1999). We identified outliers as points outside the bagplot's fence and replaced them with estimated temperatures using Friedman's super smoother (Friedman 1984). Data screening resulted in removing and estimating 3.3% of the temperature observations. We created bagplots using the R package aplpack (Wolf 2019).

### 2.2 Data Analysis

We used generalized linear mixed models (GLMM) with a logit link function (i.e., logistic regression) to assess the influence of temporal predictors (hereafter time) and environmental temperatures on the probability of turtles being on land. We chose not to combine the temporal predictors with temperature in the same model to avoid confounding factors caused by the temporal change of temperature. We treated individual turtles as random intercepts to account for individual variation and repeated measures. We standardized continuous variables to facilitate model convergence (Harrison et al. 2018). We used Akaike's information criterion corrected for small sample size (AIC*c*) to rank candidate models and considered candidate models to have strong support when ∆AIC*c* < 2 (Burnham et al. 2011).

We included sex as a candidate predictor in the time and temperature analyses based on previous studies that indicated the relationships likely differ between the sexes (e.g., Tingley et al. 2010; McCoard et al. 2016; Thompson et al. 2018; Cochrane et al. 2019). For the time analysis, we included day versus night, hour of day, day of year, week of year, month of year, and year (2015 vs. 2016) as candidate predictors. We tested both linear and quadratic relationships for the continuous variables. We created a priori candidate model set that included the null model, each variable, and ecologically relevant combinations of variables, including potential interactions.

For the temperature analysis, we first performed a preliminary analysis using a generalized additive mixed model to determine if the shape of the temperature relationships was linear or quadratic, which indicated the relationships were linear. We then performed a model selection to determine which environmental predictor (i.e., open canopy air temperature, closed canopy air temperature, stream temperature) had the greatest explanatory power. For the strongest predictor, we estimated its influence on the probability of turtles being on land using the complete data set, as well as in May and July. May represents the spring pre‐nesting activity period in our study area, where wood turtles remain close to the stream and routinely move between the two habitat classes, whereas July represents the mid‐summer post‐nesting activity period, where we would expect much greater terrestrial habitat use and increased movement away from the stream (Brown et al. 2016; Cochrane et al. 2019). To address the influence of distance from the stream, we performed an additional analysis that restricted the July data to observations within 50 m of the stream. Cochrane et al. (2019) estimated that the mean daily movement rate outside of the nesting season for these turtles was approximately 50 m, and we assumed that turtles within this distance were close enough to return to the stream if it was beneficial for thermoregulation. The restricted data analysis included 22% (*n* = 63,193 observations) and 53% (*n* = 31,693 observations) of the total observation data in July for females and males, respectively.

For the most supported models, we assessed each coefficient's direction, magnitude, and strength of the effect (85% confidence interval [CI]; Arnold 2010) and estimated the explanatory power of the model using pseudo‐*r* ^2^ (Nakagawa and Schielzeth 2013). We also compared AIC*c* ranks and pseudo‐*r* ^2^ of the most supported time and temperature models to determine which model explained aquatic–terrestrial habitat use better. We conducted GLMM analyses using Program R (R Core Team 2023) and the package lme4 (Bates et al. 2015) and performed model selections using the package aicmodavg (Mazerolle 2020). We generated model predictions using the package ggeffects (Lüdecke 2018) and created graphs using the packages ggplot2 (Wickham 2016) and ggpubr (Kassambara 2023).

## 3 Results

We recorded 242,781 observations from the 23 wood turtles tracked between May and August 2015 and 2016, and we classified 234,663 as water or land observations (Table 1). Turtles were on land for 65% of the observations, and the GPS units fixed a location for 31% of the land observations. For the time analysis, quadratic relationships were supported for all continuous variables (i.e., hour, day, and week). Every time variable was more supported than the null model, and hour was the most supported individual predictor (Table 2). Models that included interactions between time variables and sex outperformed models that did not have sex or included sex as an additive effect. The most supported model included interactions between hour, day, and sex (*w*_i_ = 1, pseudo‐*r* ^2^ = 0.56).

**TABLE 1:** Number of observations for 23 adult wood turtles ( *Glyptemys insculpta* ) that were monitored using global positioning system (GPS) trackers at 10‐min intervals in northeastern Minnesota between May and August (Aug) of 2015 and 2016, organized by sex, day versus night, location state (i.e., terrestrial and aquatic), and month. Observations with unclassified location states were excluded (*n* = 8118).

	Aquatic			Terrestrial			Total		
	Day	Night	Total	Day	Night	Total	Day	Night	Total
Female (*n* = 16)									
May	6150	9516	15,666	11,880	541	12,421	18,030	10,057	28,087
June	6693	9499	16,192	27,179	7182	34,361	33,872	16,681	50,553
July	4053	5855	9908	37,263	16,022	53,285	41,316	21,877	63,193
Aug	1067	1715	2782	10,019	5413	15,432	11,086	7128	18,214
Total	17,963	26,585	44,548	86,341	29,158	115,499	104,304	55,743	160,047
Male (*n* = 7)									
May	2277	3344	5621	5657	812	6469	7934	4156	12,090
June	3861	5404	9265	11,763	2398	14,161	15,624	7802	23,426
July	6238	7844	14,082	14,585	3026	17,611	20,823	10,870	31,693
Aug	1456	2090	3546	2999	862	3861	4455	2952	7407
Total	13,832	18,682	32,514	35,004	7098	42,102	48,836	25,780	74,616
Total (*n* = 23)									
May	8427	12,860	21,287	17,537	1353	18,890	25,964	14,213	40,177
June	10,554	14,903	25,457	38,942	9580	48,522	49,496	24,483	73,979
July	10,291	13,699	23,990	51,848	19,048	70,896	62,139	32,747	94,886
Aug	2523	3805	6328	13,018	6275	19,293	15,541	10,080	25,621
Total	31,795	45,267	77,062	121,345	36,256	157,601	153,140	81,523	234,663

**TABLE 2:** Model selection results using Akaike's information criterion corrected for small sample size (AIC_*c*_) to determine the most supported models for estimating the probability of being terrestrial for adult wood turtles ( *Glyptemys insculpta* ) in northeastern Minnesota. We used generalized linear mixed models with a logit link function and individual turtles treated as random effects. Candidate variables for the time analysis included hour, day of year (day), month of year, week of year, day or night (dn), and sex. Candidate variables for the temperature analysis included open canopy air temperature, closed canopy air temperature, and water temperature. We first determined which of the temperature variables was most supported and then the most supported variable was tested with sex. Quadratic terms are denoted with (q). Akaike weights are represented as *w*_i_. The null model is shown as (.) and included only the intercept.

Analysis	Model	Parameters	AIC_*c*_	∆AIC_*c*_	*w*_i_
Time	Hour (q) × day (q) × sex	19	217,376.6	0.00	1
	Hour (q) × day (q) + sex	11	224,982.6	7606.07	0
	Hour (q) + month × sex	11	226,800.1	9423.53	0
	Hour (q) × day (q)	6	231,218.4	13,841.83	0
	Hour (q) + month + sex	8	232,447.7	15,071.16	0
	Hour (q) + month	7	232,453.5	15,076.98	0
	Dn + week (q) × sex	8	233,437.8	16,061.20	0
	Dn + month × sex	10	235,107.9	17,731.36	0
	Dn + week (q) + sex	6	239,240.9	21,864.28	0
	Dn + week (q)	5	239,246.7	21,870.16	0
	Hour (q)	4	240,139.9	22,763.29	0
	Dn + month + sex	7	240,467.1	23,090.53	0
	Dn + month	6	240,472.9	23,096.29	0
	Dn	3	248,203.7	30,827.09	0
	Week (q)	4	272,013.7	54,637.17	0
	Day (q)	4	272,073.9	54,697.33	0
	Month	5	273,089.1	55,712.51	0
	Sex	3	279,330.1	61,953.50	0
	Year	3	279,331.1	61,954.55	0
	(.)	2	279,333.5	61,956.91	0
Temperature	Open canopy air temperature	2	229,030.8	0.00	1
	Closed canopy air temperature	2	242,984.6	13,953.86	0
	Stream temperature	2	285,167.2	56,136.46	0
	(.)	1	68,071.34	68,071.34	0
Temperature and sex	Temperature × Sex	5	213,578.3	0.00	1
	Temperature + Sex	4	213,747.3	168.97	0
	Temperature	3	213,755.8	177.45	0
	Sex	3	279,330.1	65,751.74	0
	(.)	2	279,333.5	65,755.16	0
Temperature versus time	Temperature × Sex	5	213,578.3	0.00	1
	Hour (q) × day (q) × sex	19	217,376.6	3798.24	0
	(.)	2	279,333.5	65,755.16	0

**FIGURE 3:**
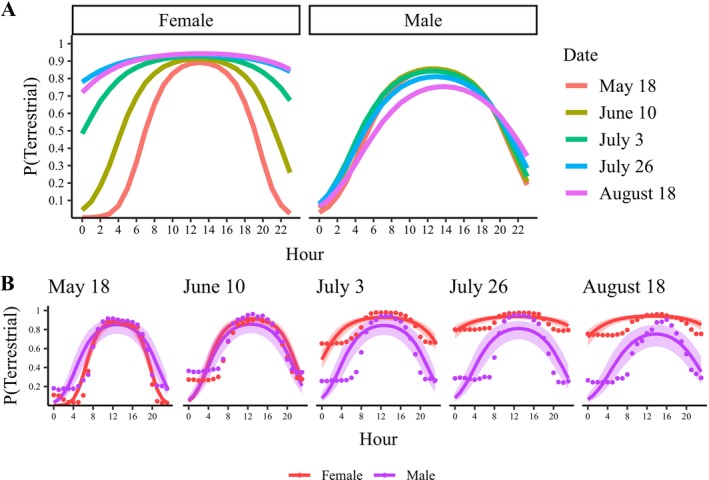
Model‐estimated relationships for the hourly probability of wood turtles ( *Glyptemys insculpta* ) being terrestrial by sex (A) and date (3‐week intervals; B) for 23 adult wood turtles monitored using global positioning system (GPS) trackers in northeastern Minnesota in 2015 and 2016. The bands represent 85% confidence intervals. The points represent the observed proportion of wood turtles in a terrestrial state during each hour of three weeks centered around the graph's date.

The most supported model estimated that the probability of being terrestrial was highest at mid‐day for males and females throughout the active period (Figure 3, Table 3). The sexes had similar activity patterns during the spring pre‐nesting activity period, with probabilities of being terrestrial < 0.1 at night and > 0.8 at mid‐day. The sexes diverged as the summer progressed, with males returning to the stream and females becoming primarily terrestrial at night. In mid‐summer (i.e., August), the probability of females being terrestrial at night was approximately 0.8, whereas for males it was < 0.1. However, according to the observation data, about 30% of males remained terrestrial overnight in August, indicating that nighttime terrestrial activity may be underestimated in the model for males (Table 1, Figure 3).

**TABLE 3:** Parameter estimates (*β*) and 85% confidence intervals (CI) for the most supported models for estimating the probability of being terrestrial for adult wood turtles ( *Glyptemys insculpta* ) in northeastern Minnesota. The most supported model for the time analysis included interactions between hour of day, day of year (day), and sex. The most supported model for the temperature analyses had an interaction between open canopy air temperature and sex. The temperature analyses estimated the influence of air temperature on habitat use for the complete dataset (May–August), May, and July (July analysis restricted to locations < 50 m from the river).

Analysis	Predicter	*β*	85% CI
Time (full dataset)	Intercept	2.522	2.235–2.810
	Hour	0.255	0.244–0.266
	Hour^2^	−0.849	−0.863 to −0.835
	Day	0.239	0.222–0.255
	Day^2^	−0.055	−0.068 to −0.041
	Sex: male	−0.849	−1.383 to −0.315
	Hour × day	−0.299	−0.311 to −0.288
	Hour × day^2^	0.158	0.149–0.166
	Hour^2^ × day	0.689	0.675–0.703
	Hour^2^ × day^2^	−0.274	−0.285 to −0.264
	Hour × sex	0.123	0.103–0.143
	Hour^2^ × sex	−0.404	−0.429 to −0.380
	Day × sex	−0.416	−0.442 to −0.391
	Day^2^ × sex	−0.043	−0.064 to −0.021
	Hour × day × sex	0.297	0.278–0.315
	Hour × day^2^ × sex	−0.086	−0.101 to −0.072
	Hour^2^ × day × sex	−0.538	−0.560 to −0.517
	Hour^2^ × day^2^ × sex	0.284	0.267–0.301
Temperature (Full Dataset)	Intercept	1.581	1.353–1.810
	Temperature	1.669	1.654–1.684
	Sex: male	−1.321	−1.722 to −0.919
	Temperature × sex	−0.214	−0.238 to −0.191
Temperature (May)	Intercept	1.425	1.161–1.689
	Temperature	2.274	2.235–2.313
	Sex: male	1.011	0.532–1.490
	Temperature × sex	0.395	0.314–0.476
Temperature (< 50 m July)	Intercept	−1.216	−1.712 to −0.720
	Temperature	1.773	1.719–1.827
	Sex: male	−1.515	−2.377 to −0.652
	Temperature × sex	0.913	0.824–1.001

**FIGURE 4:**
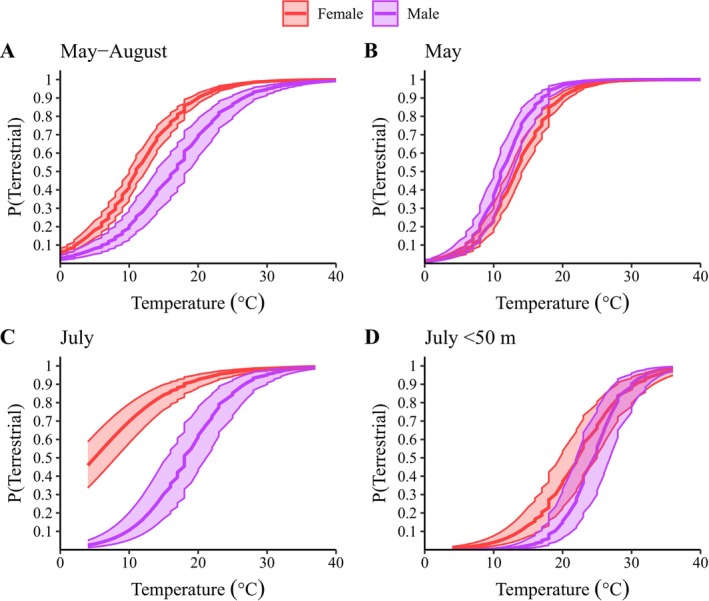
Model‐estimated influence of open canopy air temperature (°C) on the probability of adult wood turtle ( *Glyptemys insculpta* ) presence in terrestrial environments for each sex during the full study period (A), May (B), July (C), and July restricted to terrestrial locations within 50 m of the river (D). We monitored 23 wood turtles using global positioning system (GPS) trackers and collected environmental temperatures at 10‐min intervals in northeastern Minnesota between May and August 2015 and 2016. The bands represent 85% confidence intervals.

For the temperature analysis, we found that open‐canopy air temperature was a stronger predictor than closed‐canopy air temperature and stream temperature for aquatic‐terrestrial habitat use (*w*_i_ = 1). The most supported model included an interaction between temperature and sex (Table 2; *w*_i_ = 1, pseudo‐*r* ^2^ = 0.61). The model estimated that the probability of being terrestrial was greater than aquatic when open‐canopy air temperature exceeded 11°C and 17°C for females and males, respectively (Figure 4, Table 3). When the analysis was restricted to May, the probability of being terrestrial was greater than aquatic when open‐canopy air temperature exceeded 14°C and 12°C for females and males, respectively (Figure 4; Table 3, pseudo‐*r* ^2^ = 0.88). As with the time analysis, the temperature relationship for the sexes diverged as the summer progressed, with females less likely to return to the stream when air temperature decreased. However, when we restricted the July analysis to observations < 50 m from the stream, both sexes were likely to return to the stream when the air temperature dropped (Figure 4, Table 3). For the full data sets, the temperature model had more support than the time model (Table 2; *w*_i_ = 1), even with distance from the stream influencing the relationship in summer.

## 4 Discussion

Our study found that time and temperature had strong explanatory power for wood turtle aquatic‐terrestrial habitat use patterns, but temperature was a stronger predictor. This finding supports our hypothesis and previous research suggesting that thermoregulatory needs influence wood turtle movement between aquatic and terrestrial environments (Dubois et al. 2009). The strong influence of air temperature on habitat use could partially explain the substantial geographic variation in temporal activity patterns for wood turtles, such as the timing of spring emergence and winter dormancy (reviewed by Willey et al. 2021).

Our estimated time and temperature‐based habitat use patterns for the pre‐nesting activity period are congruent with previous research on wood turtle behavior, with turtles typically leaving the water during the warmest portions of the day and returning to water at night (Harding and Bloomer 1979; Arvisais et al. 2002; Dubois et al. 2009). Our temperature model estimated that we can expect nearly all turtles to be terrestrial at air temperatures > 20°C, which aligns with the temperature of peak detection probability for terrestrial population surveys in the study region (Brown et al. 2017; Staggs et al. 2024). Interestingly, detection probability for population surveys declines at high temperatures (Brown et al. 2017; Staggs et al. 2024) even though wood turtles typically remain on land as air temperature increases in our study area. We suspect the declining detection probability is caused mainly by turtles seeking cover in closed‐canopy environments at high temperatures, such as within dense shrubs or burying under leaf litter and into rotting logs, thus decreasing our probability of detecting them on land.

**FIGURE 5:**
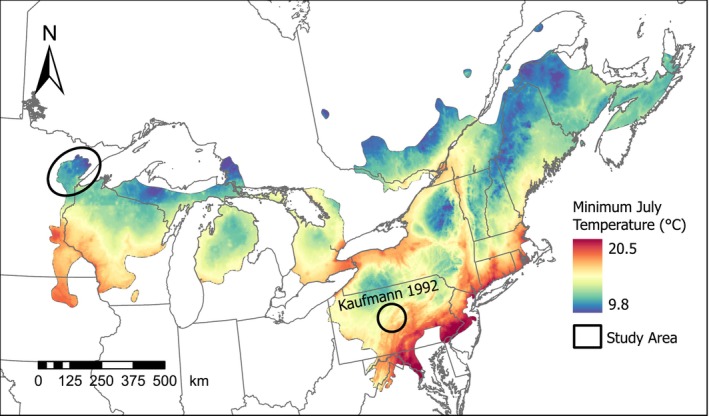
Average minimum July temperature (1991–2020; Fick and Hijmans 2017) within the geographic distribution of the wood turtle ( *Glyptemys insculpta* ; distribution adapted from Powell et al. 2016). The upper left circle indicates our study area in northeastern Minnesota, where we tracked locations and temperatures of 23 wood turtles between May and September of 2015 and 2016 to assess the influences of time and temperature on aquatic‐terrestrial habitat use. The lower right circle indicates the study area in central Pennsylvania where Kaufmann (1992) studied wood turtle aquatic‐terrestrial habitat use.

Our results were consistent with previous studies across the geographic distribution that found males were more aquatic than females during the post‐nesting activity period (Kaufmann 1992; Compton et al. 2002; Brown et al. 2016; McCoard et al. 2018). However, we expected the proportion of males returning to the stream at night to be lower than we observed (0.71), as the highest proportion reported previously was 0.51 (Kaufmann 1992). This difference may be due to differing regional climates. The Kaufmann (1992) study was conducted on a population in central Pennsylvania with a warmer climate than northeastern Minnesota (Figure 5). Thus, assuming the temperature relationship estimated in this study is applicable across the species' distribution, we would expect the probability of males returning to streams at night during summer to decrease in central Pennsylvania. However, we note that our sample size for males was small in the study (i.e., seven individuals tracked for 1–2 summers), and thus our observed proportions may not be representative of the population.

In contrast to males, we found that females rarely returned to the stream at night during the post‐nesting activity period, with an even lower proportion returning in our study than was documented in central Pennsylvania (28%; Kaufmann 1992). While there may be thermoregulatory benefits to returning to the stream at night during summer, it is not required for survival. Energy cost of travel and increased risk of predation may be reasons that female turtles do not return to the stream at night. Greater female use of inland habitat has been documented in populations across the species' distribution (e.g., Tingley et al. 2010; Brown et al. 2016; McCoard et al. 2016; Latham et al. 2023), and our results suggest there is a tradeoff between thermoregulatory benefits and energy expended to reach the stream. Further research is needed to clarify why space‐use patterns during the post‐nesting period differ between males and females, specifically to test hypotheses of male avoidance by females during the non‐breeding season and differential food resource needs. A recent study in New Brunswick, Canada found no evidence that diets differed between adult males and females based on stable isotope analyses, with both sexes predominantly consuming terrestrial invertebrates (Bellamy et al. 2023).

It is likely that wood turtles utilized aquatic habitat other than the stream (e.g., wetlands and ponds). Many studies report still‐water habitat use (Arvisais et al. 2004; Greaves 2008; Brown et al. 2016), however the differences in thermoregulatory benefits between different types of aquatic habitat are unknown. Location states could have been misclassified as terrestrial if shallow and still aquatic habitat has a warmer temperature profile relative to spring‐fed stream habitat or temperature loggers were not fully submerged in water. Therefore, non‐stream aquatic habitat use may be underestimated during the summer.

The results of our study have several conservation implications for wood turtles. State guidelines for proposed land management actions in occupied wood turtle habitat typically use seasonal space restrictions to protect the species from direct mortality, such as excluding activities within 75 m of the stream from mid‐March to mid‐May (Wisconsin Department of Natural Resources 2017). Our results indicate that during the pre‐nesting activity period, temperature‐based restrictions could allow terrestrial habitat management and restoration actions while maintaining a low probability of direct mortality. In addition, our model estimates that most turtles in a population are unlikely to be available for detection during spring terrestrial population surveys when air temperature is < 10°C. Managers could use our results to define a minimum temperature threshold for performing terrestrial surveys to determine wood turtle presence or monitor population health. However, this study only considered adult wood turtles and thus the results cannot be used to predict juvenile habitat use. For future studies on this topic, it would be beneficial to include smaller individuals and to quantify how body size interacts with temperature to influence aquatic‐terrestrial habitat use. Finally, the strong influence of air temperature on daily aquatic‐terrestrial activity patterns suggests that terrestrial activity will likely increase in our study area as the climate warms (Handler et al. 2014). This climate change response could negatively impact adult survivorship rates in the region by increasing interactions with terrestrial mesopredators and anthropogenic threats such as road mortality and illegal collection (Levell 2000; Lapin et al. 2019).

## Author Contributions

**Jena M. Staggs:** conceptualization (supporting), data curation (lead), formal analysis (lead), investigation (equal), methodology (equal), validation (lead), visualization (lead), writing – original draft (lead), writing – review and editing (supporting). **Donald J. Brown:** conceptualization (equal), funding acquisition (equal), investigation (equal), methodology (equal), project administration (equal), supervision (equal), writing – original draft (supporting), writing – review and editing (lead). **Madaline M. Cochrane:** conceptualization (equal), data curation (equal), investigation (equal), methodology (equal), writing – review and editing (supporting). **Andrew F. Badje:** funding acquisition (equal), project administration (equal), supervision (supporting), writing – review and editing (supporting). **Ron A. Moen:** conceptualization (equal), funding acquisition (equal), investigation (equal), methodology (equal), project administration (equal), supervision (equal), writing – review and editing (supporting).

## Conflicts of Interest

The authors declare no conflicts of interest.

## Data Availability

Data used for this study and code to reproduce results are available through the Dryad data repository: https://doi.org/10.5061/dryad.cvdncjthb.
